# Colorectal Cancer Surveillance in Patients with Inflammatory Bowel Diseases: Chromoendoscopy or Non-Chromoendoscopy, That Is the Question

**DOI:** 10.3390/jcm11030509

**Published:** 2022-01-20

**Authors:** Roberto Gabbiadini, Ferdinando D’Amico, Alessandro De Marco, Maria Terrin, Alessandra Zilli, Federica Furfaro, Mariangela Allocca, Gionata Fiorino, Silvio Danese

**Affiliations:** 1 Department of Biomedical Sciences, Humanitas University, Via Rita Levi Montalcini 4, 20072 Pieve Emanuele, Milan, Italy; roberto.gabbiadini@humanitas.it (R.G.); ferdinando.damico@humanitas.it (F.D.); alessandro.demarco@humanitas.it (A.D.M.); maria.terrin@humanitas.it (M.T.); 2 Gastroenterology and Endoscopy, IRCCS Ospedale San Raffaele and Vita-Salute San Raffaele University, 20132 Milan, Italy; zilli.alessandra@hsr.it (A.Z.); allocca.mariangela@hsr.it (M.A.); fiorino.gionata@hsr.it (G.F.); 3 IBD Center, IRCCS Humanitas Research Hospital, Via Manzoni 56, 20089 Rozzano, Milan, Italy; federica.furfaro@humanitas.it

**Keywords:** inflammatory bowel disease, CRC surveillance, high-definition endoscopy, chromoendoscopy, random biopsies

## Abstract

Subjects affected by ulcerative colitis and Crohn’s disease with colonic localization have an increased risk of colorectal cancer (CRC). Surveillance colonoscopy is recommended by international guidelines as it can detect early-stage CRC. Based on previous evidence, in 2015 the Surveillance for Colorectal Endoscopic Neoplasia Detection and Management in Inflammatory Bowel Disease Patients International Consensus indicated dye chromoendoscopy (DCE) as the most effective technique for detecting dysplasia. However, advances in endoscopic technology such as high-definition colonoscopes and dye-less virtual chromoendoscopy (VCE) may change future practice. In this review, we summarize the available evidence on CRC surveillance in IBD, focusing on the emerging role of high-definition white light endoscopy (HD-WLE) and VCE over the standard DCE, and the current role of random biopsies.

## 1. Introduction

**Figure 1:**
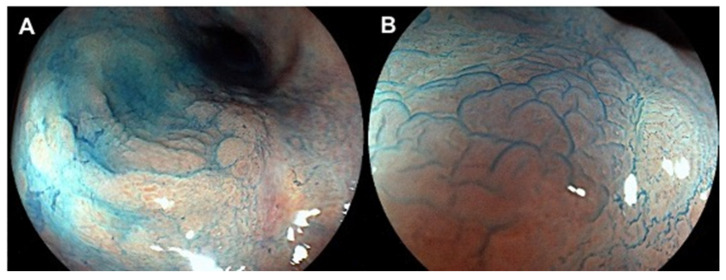
(**A**) High-definition dye-chromoendoscopy with methylene blue in a male patients with ulcerative colitis highlighting a non-polypoid lesion of the sigma-rectum junction. (**B**) Enlarged image showing edematous mucosa and the lengthening of the crypts. Histopathological staging after surgery: high-grade dysplasia.

Inflammatory bowel diseases (IBD), which include ulcerative colitis (UC) and Crohn’s disease (CD), are life-long disorders characterized by chronic relapsing inflammation of the gastrointestinal tract [1,2]. IBD are a global burden, with higher prevalence in Europe and North America and a rapidly increasing incidence in newly industrialized countries [3,4]. The etiology of IBD remains mostly unclear. Studies suggest a multifactorial pathogenesis including genetic susceptibility, abnormal intestinal microbiota, different environmental factors, and immunological alterations leading to an irregular and persistent inflammatory response [5,6]. The chronic inflammatory stimulation of the colonic mucosa increases the risk of developing dysplasia and colorectal cancer (CRC) in subjects affected by IBD [7]. Indeed, the risk of CRC in IBD patients is 1.5–2 times greater than general population [8], with a reported incidence of CRC that ranges from 41.5/100,000 person-years to 543.5/100,000 person-years (py) [9]. In particular, the incidence in CD ranges from 19.5 to 344.9/100,000 py, while in UC the incidence rate varies from 54.5 to 543.5/100,000 py [9]. Furthermore, both UC and CD have a higher CRC-associated mortality [10]. Colonoscopy surveillance can detect early-stage CRC in subjects with IBD, thus decreasing CRC development and CRC-associated mortality [11]. Therefore, a CRC surveillance program is recommended by several international guidelines, generally after 8–10 years from disease onset [12,13,14,15], even if some guidelines suggest a more cautious timing (after six to eight years) [16], as some studies showed that a significant proportion of CRC could develop prior to eight years of disease [17,18]. A successive interval of one to five years is then established on the basis of patient and disease risk factors [13]. Indeed, subjects displaying high-risk characteristics (i.e., stricture, primary sclerosing cholangitis) should undergo surveillance colonoscopy every year, while patients with intermediate risk features (i.e., post-inflammatory polyps, family history of CRC) can be checked every two to three years. On the other hand, if no risk factors for CRC are present, the surveillance interval can be extended to five years [13]. The approach to CRC surveillance in IBD is continuously evolving due to the expanding advance in endoscopic technology, and the debate is still ongoing about the best method to detect dysplasia and CRC. Historically, the traditional technique used to perform surveillance consisted in standard definition white light endoscopy (SD-WLE) with multiple random biopsies (random 4 quadrant biopsies every 10 cm for a minimum of 32) plus targeted biopsies of visible lesions [19]. Due to the suboptimal quality image of the previous technology, dysplasia was not easily visible (or “invisible”) and the majority of colonic dysplastic lesions were detected by nontargeted biopsies [12,19,20]. Dye chromoendoscopy (DCE) with target biopsies has been proposed as an innovative methodology for detecting dysplasia, overcoming SD-WLE shortcomings. During DCE, the physician applies a contrast agent such as indigo carmine or methylene blue to the colon epithelium providing contrast enhancement and highlighting the poorly visible lesions of the mucosa [20,21] (Figure 1). Since its first use in a randomized controlled trial for early detection of intraepithelial neoplasia in UC [22], various meta-analysis showed that DCE had a higher diagnostic yield of dysplastic lesions than SD-WLE (incremental yield of 7% on a per patient basis, 95% CI 3.2–11.3) [20,23]. Consequently, the Surveillance for Colorectal Endoscopic Neoplasia Detection and Management in Inflammatory Bowel Disease Patients: International Consensus (SCENIC) recommended to use DCE over SD-WLE when performing surveillance [20]. However, SD-WLE is no longer sufficient for CRC surveillance due to the development of high-definition white light endoscopy (HD-WLE). High-resolution equipment offers a wider field of vision, a higher pixel density, and faster line scanning on the monitor, producing sharper images with fewer artifacts [24,25], leading to an improved targeted detection of dysplastic lesions [26].These advances and findings have questioned whether DCE may offer a significant advantage in dysplasia detection only when compared to SD-WLE and not to HD-WLE. Indeed, growing evidence shows that in the near future, HD systems may achieve an equivalent dysplasia detection yield without the addition of pan-colonic dye spray [24], and that invisible dysplasia may be only a consequence of the less quality image of SD-WLE [27]. Furthermore, the scenario of CRC surveillance in IBD has become more tricky with the development of the dye-less, virtual chromoendoscopy (VCE). This technology has emerged as a valid contrast enhancement system without dye application, thus being less time-consuming and less expensive than DCE [28,29]. By simply pushing a button, VCE provides an instant digital staining, enhancing colonic mucosal details and vascularization [24,29]. Such groundbreaking novelties have risen concern about the position of HD-WLE, DCE, or VCE as the future preferred method for surveillance [29] and about the benefit of random biopsies in this era of constant advancing image technology [20]. In the last years, accumulating evidence on this topic have been produced with heterogeneous results [30,31]. In this review, we aimed to summarize the available evidence in the continuous expanding scenario of CRC surveillance in IBD since the SCENIC consensus published in 2015, focusing on the emerging role of HD-WLE and VCE over the standard DCE, and the current role of random biopsies.

## 2. Virtual Chromoendoscopy: Technical Aspects

**Figure 2:**
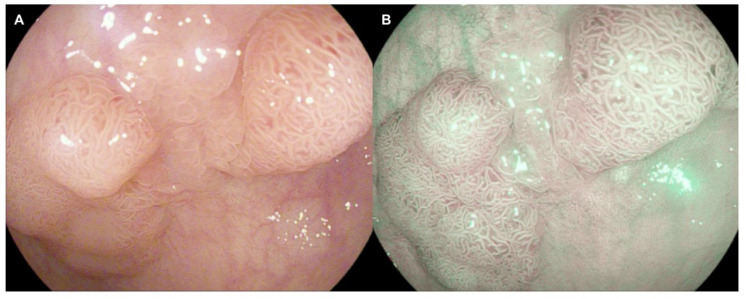
Large polypoid lesion of the sigma in a female patient with Crohn’s disease, pit pattern IIIL based on Kudo classification. (**A**) High-definition white light endoscopy. (**B**) High-definition virtual chromoendoscopy with i-SCAN 3. Histopathological staging after endoscopic resection: low-grade dysplasia.

Various companies equipped their colonoscopes with VCE technology in order to enhance details of the colonic mucosa without using further equipment. Narrow-band imaging (NBI, Olympus, Tokyo, Japan) was introduced in 2005. It is a blue-light technology that improves visualization of superficial mucosal structures, particularly superficial microvessels, by filtering the illumination light to wavelengths which are absorbed by hemoglobin [32,33]. Flexible spectral imaging color enhancement (FICE, Fujifilm, Tokyo, Japan) is a post-processor application which enhances vascularization and colonic mucosa images. This technology chooses only specific wavelengths from the white-light image and reconstructs a composite color-enhanced image [33]. Also, iSCAN (Pentax, Tokyo, Japan) is a post-processing image enhancement technology that produces digital contrast for a more defined mucosal pattern and vascularization. Three iSCAN modes are available. iSCAN 1 uses surface-enhancement (SE) plus contrast-enhancement (CE) technologies and is recommended for detection. iSCAN 2 is a combination of SE and tone enhancement (TE) technologies and is suggested for lesion characterization. iSCAN 3 comprises SE, CE, and TE and is recommended for lesion delineation [33,34] (Figure 2). Linked color imaging (LCI, Fujifilm, Japan) has been developed as a new pre-process image-enhanced endoscopy which differentiates the red color spectrum better than white-light imaging, making lesions more reddish and the nearby mucosa more whitish, thus achieving an improved contrast for identifying colonic alterations [35,36]. Blue Light Imaging (BLI, Fujifilm, Japan) is a VCE based on the direct emission of blue light using a short wavelength (410 nm) which is specifically absorbed by haemoglobin producing bright, high-intensity contrast imaging that might increase optical diagnosis and adenoma detection [37,38].

## 3. Methods

We conducted a literature search in the PubMed, Embase, and Scopus databases. The keywords used were “Crohn’s disease”, “CD”, “ulcerative colitis”, “UC”, “inflammatory bowel disease”, “IBD”, “surveillance”, “colorectal cancer”, “CRC”, “dysplasia”, “chromoendoscopy”, “virtual chromoendoscopy”, “dye chromoendoscopy”, “high definition endoscopy”, “random biopsies”, and “targeted biopsies”. We selected all relevant full text papers published since the SCENIC consensus up to October 2021 that used high-definition colonoscopes. Additional articles were screened from the reference list of the selected papers.

## 4. Results

### 4.1. DCE vs. HD-WLE

A prospective randomized trial by Wan et al. [39] compared the dysplasia-detection rate between DCE with targeted biopsies (CET), HD-WLE with targeted biopsies (WLT), and HD-WLE with random biopsies (WLR) in 122 UC patients undergoing 447 colonoscopies. WLR and CET examinations displayed a similar detection rate that was better than WLT (respectively 8.1% and 9.7% vs. 1.9%; *p* = 0.014 and 0.004). Nevertheless, during a long-term follow-up (>3 years) CET detected better than both WLT (13.3% vs. 1.6%, *p* = 0.015) and WLR (13.3% vs. 4.9%, *p* = 0.107) [39]. The superiority of DCE over HD-WLE in the field of surveillance in IBD was also observed in the single-centre randomized, controlled trial (RCT) by Alexanderson et al. [40]. In this study, 305 UC or CD patients were assigned to DCE (*n* = 152) or HD-WLE (*n* = 153), each arm performing both random and targeted biopsies. DCE showed a higher detection of dysplasia compared to HD-WLE (17 vs. 7; *p* = 0.032) [40]. Even in a retrospective study by Kim et al. DCE performed better than HD-WLE in dysplasia detection [41]. In this study, a paired comparison between 159 DCE and 131 WLE controls (of which 124 HD-WLE and 7 SD-WLE) was performed. A higher number of both polypoid and non-polypoid lesions was found in DCE group compared with WLE. The overall neoplasia detection rate was 40.9% in the DCE group and 23.7% in WLE (*p* = 0.002). Interestingly, these results did not change significantly even after excluding the 7 SD-WLE procedures [41]. Similarly, another retrospective study conducted by Sekra et al. [42] showed that DCE with targeted biopsies was associated with a higher nonpolypoid dysplasia detection rate compared to HD-WLE. One hundred and ten surveillance exams were performed (76 HD-WLE, 34 DCE), and seven nonpolypoid dysplastic lesions were detected, all with DCE. On the other hand, the polypoid dysplasia detection rate was similar in both techniques (*p* = 0.12) [42]. Furthermore, some studies observed a higher detection rate of DCE also when performing the colonoscopy surveillance with dye in the same session or soon after WLE. Indeed, Deepak and colleagues observed that performing DCE in patients with a history of dysplasia on an index WLE could identify new lesions previously not seen [43]. Of the 95 patients with dysplasia discovered on the index WLE (55 found on targeted biopsies and 40 on random biopsies), the first subsequent DCE identified dysplastic lesions in 50 cases, of which 34 were new lesions, suggesting the use of DCE in this high-risk setting [43]. Similarly, in a prospective multicentre cohort study by Carballal et al., DCE exhibited a 57.4% incremental yield of dysplasia compared to WLE [44]. Each colonic tract was first examined with WLE and then with indigo carmine CE in the same exam. This result remained similar when SD-WLE and HD-WLE were considered separately (respectively 41.5% and 58.5% of total procedures) [44]. Somewhat differently, a recent study by Coelho-Prabhu et al. found a comparable dysplasia diagnostic yield between DCE and HD-WLE in subjects affected by IBD involving the colon [45]. In this retrospective observational cohort study, 808 colonoscopies were carried out, including 150 procedures (18.6%) with DCE. Polypoid dysplasia was detected in 50 patients (33.0%) in the DCE group and in 79 patients (12.0%) in the HD-WLE group (*p* < 0.01). Dysplasia in random biopsies was observed in 15 subjects (10%) during DCE and 24 subjects (3.6%) during HD-WLE (*p* < 0.001). However, when considering for other dysplasia risk factors at multivariate analysis (i.e., older age at diagnosis, endoscopist expert in IBD, endoscopist with <10 years’ experience, prior random dysplasia, primary sclerosing cholangitis), the detection of both polypoid and random dysplasia between DCE and HD-WLE did not differ [45]. Similarly, in a large retrospective study by Moojweer et al. in which 440 DCE procedures were compared with 1802 WLE procedures with random and targeted biopsies, the dysplasia detection between the two methodologies was similar (11% in the DCE group and 10% in WLE group; *p* = 0.80) [46]. Furthermore, these results were also confirmed when taking into account only targeted biopsies in the two techniques (*p* = 0.30). CRC risk factors were similar in both categories except for more subjects with CD extensive colitis and with first-degree relative with CRC in the DCE group. Nevertheless, it is important to consider that the period study was between 2000 and 2013 and different types of colonoscopes were used, not specifying how many endoscopic exams were performed with HD in the WLE group, while DCE was performed in the recent years with probably better endoscopes [46]. Another retrospective matched case-control study found no significant differences in dysplasia surveillance between DCE and HD-WLE [47]. One hundred eighty-seven IBD patients underwent colonoscopy for dysplasia surveillance (98 DCE, 89 HD-WLE). No significant difference was observed in the detection of dysplastic lesions between DCE and HD-WLE in both univariate analysis (10.2% vs. 6.7%, *p* = 0.39) and multivariate analysis, which were adjusted for age, sex, duration and type of IBD, and history of dysplasia (OR 0.91, 95% CI 0.15–5.67, *p* = 0.92), supporting that extensive use of DCE for CRC surveillance in everyday IBD clinical practice displays low cost-effectiveness [47]. In addition, several prospective RCT confirmed these results. Iacucci et al. found no significant difference in dysplasia detection between HD-WLE and DCE [48]. In this randomized trial, 270 subjects with longstanding UC undergoing surveillance colonoscopy were assigned to HD-WLE (*n* = 90), DCE (*n* = 90), and VCE using iSCAN (*n* = 90). Dysplasia (polypoid and non-polypoid) and CRC detection rates between the three techniques were comparable (HD-WLE 18.9%, DCE 17.8%, VCE 11.1%; *p* = 0.91) [48]. Another multicentre prospective RCT of 210 patients with long-standing UC conducted by Yang DH et al. [49] found that DCE with targeted biopsy was not significantly different from HD-WLE with random plus targeted biopsy for identifying colitis-associated dysplasia (CAD) (respectively 3.9% vs. 5.6%; *p* = 0.749). However, although not statistically significant, DCE showed a tendency for higher detection of CRC than HD-WLE (20.6% vs. 12.0%, *p* = 0.093) [49]. Table 1 summarizes the above mentioned studies. 

### 4.2. VCE

There is an expanding growth of data about the performance of VCE in the CRC surveillance in the field of IBD (Table 2). In a prospective multicenter study by Leifeld et al., 159 subjects affected by long-standing UC underwent two colonoscopies (one with HD-WLE and one with VCE using NBI) in a randomized sequence in a period between three weeks and three months [50]. During HD-WLE, four random biopsies every 10 cm (stepwise biopsies), two segmental random biopsies in 5 tract (segmental biopsies), and targeted biopsies were performed. During VCE using NBI, segmental and targeted biopsies were carried out. Overall, VCE with targeted plus segmental biopsies and HD-WLE with targeted plus stepwise biopsies displayed a similar intraepithelial neoplasia detection rate (NBI: 31 vs. HD-WLE 26, *p* = 0.888), but VCE collected less biopsies (NBI 11.9 vs. HD-WLE 38.6, *p* < 0.001) and took less time to withdraw (NBI 13 min vs. HD-WLE 23 min, *p* < 0.001). Furthermore, even though not statistically significant, NBI exhibited a trend in the direction of a higher detection rate (1.6 times) of targeted biopsies [50]. Similar results were observed in a multicentre RCT in which 188 patients with long standing UC or CD colitis were randomized 1:1 to undergo surveillance colonoscopy either with VCE (i-scan OE mode 2) or HD-WLE performing targeted and random biopsies in each arm [51]. No difference was observed in the neoplasia detection (VCE 14.9% vs. HD-WLE 24.2%; *p* = 0.14) and withdrawal time (VCE 25.5 min vs. HD-WLE 24 min, *p* = 0.216) between the two techniques. In addition, the yield of random biopsies was considerably low. Overall, 6751 random biopsies of the colon identified one neoplasia (low grade dysplasia with active background disease) [51]. VCE has also been compared to DCE in several studies. A multicentre RCT including 131 long-standing UC showed no significant difference for the detection of CAD between DCE with methylene blue (*n* = 66) and VCE with NBI (*n* = 65) [respectively 21.2% vs. 21.5%; odd ratio 1.02 (95% CI 0.44 to 2.35, *p* = 0.964)] [52]. Furthermore, the withdrawal time was significantly shorter in the NBI arm (NBI 18.5 min vs. DCE 27.0 min, *p* < 0.001), even after clustering the patients according to the whole number of biopsies obtained during the exam [52]. Also VCE using FICE, in a randomized delayed crossover trial by Gulati et al., showed a dysplasia detection rate no lower than DCE with indigo carmine [53]. Forty-eight IBD candidates to CRC surveillance underwent either DCE or VCE as index colonoscopy and, after three to eight weeks, repeated colonoscopy with the other method. The diagnostic accuracy for the endoscopic diagnosis of dysplasia applying DCE or VCE was 76.9% vs. 93.7%, respectively, with DCE missing two dysplastic lesions (18.2%) and VCE missing one dysplastic lesion (9.1%) [odds ratio 2.0 (95% CI 0.10 to 118.0)] [53]. These findings were also confirmed in a prospective study conducted by González-Bernardo et al [54]. One hundred twenty-nine patients with long standing IBD were enrolled and randomized to receive either DCE (*n* = 67) or VCE using the iSCAN 1 system (*n* = 62). All endoscopic exams were performed by the same expert physician. The rates of detection of neoplastic lesions were similar between the two groups (DCE 17.9% vs. VCE 11.3%; *p* = 0.2). Similarly, no differences were observed also in the detection of all lesions, neoplastic or non-neoplastic. On the other hand, VCE exhibited a lower withdrawal time compared to DCE (10 vs. 14 min, respectively; *p* < 0.001) [54]. Similarly, a recent retrospective case-control study observed a comparable colonic dysplasia detection among DCE with indigo carmine and VCE with iSCAN (twin-mode 1–3) in subjects with colonic IBD [55]. DCE was performed in 98 patients, while VCE was performed in 93 patients. No significant differences were observed in the per lesion (*p* = 0.526) and per patient analysis (*p* = 0.647). Even in this retrospective analysis, VCE displayed a reduced exploration time (VCE 9 min vs. DCE 14 min, *p* < 0.001) [55]. Finally, to the best of our knowledge, no studies exploring VCE using LCI or BLI in the IBD surveillance program have been published.

### 4.3. Random Biopsies in the Era of HD

The role of random biopsies IBD-CRC surveillance in the era of HD is contradictory due to improved detection of subtle colonic dysplastic lesions. In the retrospective study by Bopanna et al. [56], 28 subject affected by UC with associated high-risk factors for CRC (26 pancolitis with disease duration >15 years, two UC with primary sclerosing cholangitis), underwent surveillance HD-WLE with random biopsies every 10 cm. Overall, 924 biopsies were obtained, showing no dysplasia in any sample with only seven indefinite for dysplasia (0.7%) [56]. In a retrospective study by Gasia et al., 454 IBD patients who underwent surveillance colonoscopy were included to investigate the most effective endoscopic technique for CRC surveillance (SD-WLE, HD-WLE, VCE with iSCAN, or DCE; random plus targeted biopsies or only targeted biopsies were acquired) [57]. Interestingly, in the random biopsies group (*n* = 318, 126 with SD and 192 with HD), 32 colonic neoplastic lesions were identified, and only three lesions (9.3%) were detected exclusively by random biopsies without any visible alterations of the mucosa. Furthermore, even after excluding SD-WLE from the analysis, the targeted biopsies group showed a higher performance in the detection of neoplastic lesions (19.1% targeted biopsies vs. 10.4% random biopsies; *p* = 0.02) [57]. Accordingly, in a multicenter RCT by Watanabe et al. in which HD-WLE was applied in the majority of cases, it has been shown that targeted biopsies could detect a similar amount of neoplasia compared to random plus targeted biopsies (11.4% vs. 9.3%, respectively; *p* = 0.617) [58]. Furthermore, the percentage of dysplasia among the collected tissue samples was superior in the target arm [6.9% (24 of 350)] than in the random plus target arm [0.5% (18 of 3725)] (*p* < 0.001). Thus, the authors concluded that surveillance with only targeted biopsies could emerge as a more cost-effective strategy [58]. These results were consolidated by a subsequent retrospective cohort study based on the follow-up data of this RCT demonstrating the long-term effectiveness of targeted biopsies [59]. Indeed, no death by CRC was observed in both arms with a median 8.8-year follow-up. In addition, the incidence of advanced neoplasia was similar in each group, and the likelihood of developing high grade dysplasia/CRC in subjects characterized by a negative colonoscopy was low. Once more, the authors suggested targeted biopsy over random biopsies in real-life settings [59]. However, Moussata et al. demonstrated that random biopsies, despite their low yield, may still be useful when associated with DCE [60]. Indeed, this large prospective study which included 1000 patients with IBD that underwent surveillance DCE reported a low yield of random biopsies that was assessed at 0.2% per biopsy (68/31865). Nevertheless, random biopsies identified dysplasia in 12 out of 94 patients (12.8%) with dysplasia. Furthermore, factors like personal history of neoplasia, tubular appearing colon and PSC were independently associated with the detection of colonic dysplasia by random biopsies suggesting that they can be still used during DCE in patients with these high-risk features [60]. Along these lines, in a retrospective study of 442 examinations, Hu et al. also observed that random biopsies could be useful in increasing the diagnostic yield of CRC surveillance colonoscopies in a selected set of patients [61]. In particular, features such as longer disease duration, active inflammation, and PSC were independent risk factors for dysplasia detection on random biopsies, thus confirming that a subset of increased risk patients might benefit from random biopsies in surveillance colonoscopies [61]. Table 3 summarizes the aforementioned studies.

## 5. Discussion

Since the SCENIC consensus, an exponential growth of data has been published in the field of dysplasia surveillance in IBD. However, studies and meta-analyses produced conflicting and heterogeneous results [21,62,63,64,65,66,67,68,69,70,71,72]. Hence, to date there is no strong agreement on the best routine strategy, and this remains an unsolved topic in IBD. The turning point that probably narrowed the gap of the detection yield between DCE and WLE is the introduction of HD system [64]. Indeed, the advent of HD colonoscopy raised questions about the redundancy of DCE due the fact that the majority of dysplasia is visible with HD [27,73]. DCE also displays the limits of a longer examination time, the need for supplementary training, and may be considered impractical by the physicians [73]. Furthermore, a good quality surveillance with DCE demands an optimal view of the colonic mucosa [74], which is frequently affected by the quality of bowel preparation [75]. After the SCENIC consensus, two RCT and three retrospective studies observed a similar detection rate between DCE and HD-WLE [45,46,47,48,49]. Additionally, in recent years, two meta-analysis and three network meta-analysis agreed that DCE may add a benefit over WLE only in the setting of SD but not of HD systems, confirming the necessity of the latter technology when performing surveillance colonoscopy [21,63,64,68,69]. However, other recent studies have conversely shown an incremental yield of DCE over HD-WLE [39,40,41,42,43,44]. Therefore, discontinuing DCE during surveillance should be carefully evaluated and further RCT are needed. In the near future, surveillance with HD-WLE may be enough for patients with average risk, since a meticulous colonic mucosa examination is probably what is most important [76]. Furthermore, VCE is increasingly being proposed as an effective alternative surveillance technique in IBD. After the SCENIC consensus, four RCT exhibited a similar dysplasia detection rate between VCE and DCE with a shorter examination time in the VCE arm in most of the trials [48,52,53,54]. Interestingly, different virtual chromoendoscopy methods (iSCAN, NBI, FICE) were used, and all of them achieved similar results. Thus, differently from the SCENIC consensus, the recent guidelines published by the European Society of Gastrointestinal Endoscopy (ESGE) strongly recommend an equivalent use of DCE or VCE when performing surveillance in IBD [31]. However, competence in neoplasia detection is recommended for this purpose. Indeed, ESGE suggests self-learning by performing at least 20 pan-chromoendoscopies with at least 20 targeted biopsies with histological report [77]. To date, none of the aforementioned VCE techniques can be recommended over the other ones. RCTs directly comparing the different VCE will better define their role in the CRC surveillance program. In addition, a novel groundbreaking and promising technology such as artificial intelligence (AI) may further revolutionize the IBD surveillance colonoscopies [78,79]. This real-time computer-aided diagnosis system can help the endoscopist due to its ability to identify the lesions during the examination by flagging the suspicious colonic alteration with an optical and acoustic alert. A recent meta-analysis showed an improvement of the detection of colorectal neoplasia in a non-IBD setting [80]. However, this technology has not been applied for IBD colonic lesions yet. The magnitude of this new technology may mitigate the advantage of HD systems in the near future [79]. The role of random biopsies in the surveillance program with the HD system is another unsettled issue. Random biopsy protocols have been supported in the past, assuming the discovery of non-visible dysplasia with 90% confidence if present in 5% of the colon [27]. The SCENIC consensus demonstrated that only one out of 1000 random biopsies detects dysplasia with HD system. Furthermore, in only 1% to 1.5% subjects undergoing HD surveillance dysplasia would not be detected without performing random biopsies, differently from the SD system where 20% of dysplasia cases were discovered only by random biopsies. Thus, the panelists did not reach consensus about random biopsies [20]. Subsequent studies confirmed the low yield of random biopsies [51,56,57,58]. Interestingly, these findings were corroborated by the retrospective study of Hata et al., where HD-WLE with only targeted biopsies have proven to be as effective over the long-term as targeted plus random biopsies with no death from CRC in both groups with median 8.8-year follow-up [59]. In addition, a cost-effectiveness analysis by Konijeti et al. found that DCE with targeted biopsies was more effective and less costly than WLE with random biopsies. DCE was the most cost effective approach at sensitivity levels >23 for dysplasia detection and cost <$2200, despite the level of sensitivity of WLE for dysplasia identification [81]. However, prudence must be used in quitting random biopsies, as two recent large studies demonstrated they could improve the diagnostic yield of dysplasia and that the detection of dysplasia on random biopsies was associated with features such as PSC, tubular appearing colon, personal history of neoplasia, longer disease duration, and active inflammation. Therefore, it may be worthwhile continuing performing random biopsies in subjects displaying these risk factors.

## 6. Conclusions

Many studies on CRC surveillance in IBD are available after the SCENIC consensus. Promising data demonstrated that VCE is comparable to DCE, reducing the examination time and overcoming the need for additional equipment. RCTs comparing DCE and HD-WLE exhibited contradictory results, thus the role of HD-WLE with targeted biopsies still remains a matter of debate. Random biopsies display a low dysplasia yield; however, evidence suggests that they may be useful in a set of high-risk subjects with symptoms such as concomitant PSC, tubular colon, personal history of neoplasia, longer disease duration, and active inflammation.

## Figures and Tables

**Table 1:** Studies comparing surveillance colonoscopy using white light endoscopy or dye chromoendoscopy.

Authors	Study Design	Methods	Colonoscope Technique	Results
Wan et al. [39]	Multi-center prospective randomized controlled trial	122 UC with 447 colonoscopies. Randomization 1:1:1 to: HD-WLE with targeted biopsies (WLT) (*n* = 43) HD-WLE with random biopsies (WLR) (*n* = 40) HD-DCE with targeted biopsies (CET) (*n* = 39)	WLE vs. DCE with methylene blue	WLR and CET had more examinations that detected dysplasia than WLT (8.1%, 9.7% vs. 1.9%; *p* = 0.014 and 0.004). During a long-term follow-up (>36 months), CET exhibited more exams with dysplasia detection than WLT (13.3% vs. 1.6%, *p* = 0.015)
Alexandersson et al. [40]	Single-center prospective randomized controlled trial	305 IBD. Randomization 1:1 to: HD-WLE with targeted plus random biopsies (*n* = 153) HD-DCE with targeted plus random biopsies (*n* = 152)	WLE vs. DCE with indigo carmine	DCE identified more colonic dysplasia than HD-WLE (17 vs. 7, *p* = 0.032)
Kim et al. [41]	Single-center retrospective study	98 IBD with 290 colonoscopies. Comparison of HD-DCE (*n* = 159) vs. WLE (HD *n* = 124, SD *n* = 7)	WLE vs. DCE with methylene blue or indigo carmine	DCE achieved a higher dysplasia diagnostic yield compared to WLE (40.9% vs. 23.7%, *p* = 0.002). DCE identified a higher number of both polypoid and non-polypoid lesions than WLE.
Sekra et al. [42]	Single-center retrospective cohort study	110 IBD. Comparison of HD-DCE with targeted biopsies (*n* = 34) vs. HD-WLE with targeted plus random biopsies (*n* = 76)	WLE vs. DCE with methylene blue or indigo carmine	DCE detected nonpolypoid dysplasia in 11.8% patients while HD-WLE did not identified any dysplastic lesion (risk difference 11.8, 95% CI 0.9–22.6, *p* = 0.008). No difference were observed in the polypoid dysplasia detection rate (*p* = 0.12) between the two techniques.
Deepak et al. [43]	Multi-center retrospective cohort study	95 IBD. Subjects with dysplasia on index WLE who subsequently underwent CE	WLE vs. DCE with indigo carmine	95 patients had an index WLE with dysplasia (55 found on targeted biopsies and 40 on random biopsies). DCE displayed a higher likelihood to identify flat dysplasia compared to WLE (OR 19.3, 95% CI 9.5–39.3).
Carballal et al. [44]	Multi-centre prospective cohort study	350 IBD. Comparison of WLE (SD-WLE 41.5%, HD-WLE 58.5%) and DCE performed in the same procedure.	WLE vs. DCE with indigo carmine	94 dysplastic lesions were identified. WLE missed 40/94 dysplastic lesions with a 57.4% incremental yield for DCE. The incremental diagnostic yield was similar in SD and HD-WLE (51.5% vs. 52.3%, *p* = 0.30).
Coelho-Prabhu et al. [45]	Single-center retrospective cohort study	808 IBD. Comparison of HD-WLE with targeted plus random biopsies (*n* = 658) vs. HD-DCE with targeted plus random biopsies (*n* = 150).	WLE vs. DCE with indigo carmine	Polypoid dysplasia and dysplasia on random biopsies were both higher in DCE than HD-WLE (Polypoid: 33.0% vs. 12.0% respectively, *p* < 0.01. Random: 10% vs. 3.6% respectively, *p* < 0.001). Adjustment for dysplasia risk factors revealed a similar diagnostic yield between the two techniques.
Mooiweer et al. [46]	Multi-center retrospective study	2242 IBD. Comparison of DCE with targeted biopsies (*n* = 440) vs. WLE with targeted plus random biopsies (*n* = 1802).	WLE vs. DCE with methylene blue or indigo carmine	Dysplasia detection rate was similar in each group (DCE 11% vs. WLE 10%, *p* = 0.80). Targeted biopsies displayed a comparable dysplasia diagnostic yield in both techniques (*p* = 0.30).
Clarke et al. [47]	Single-center retrospective case-control study	187 IBD. Comparison of HD-DCE (*n* = 98) vs. HD-WLE (*n* = 89).	WLE vs. DCE with methylene blue or indigo carmine	Dysplastic lesions detection rate was not significantly different between DCE and HD-WLE (10.2% vs. 6.7% respectively, *p* = 0.39).
Iacucci et al. [48]	Single-center randomized prospective trial	270 IBD. Randomization 1:1:1 to: HD-DCE (*n* = 90) HD-VCE (*n* = 90) HD-WLE (*n* = 90)	WLE vs. DCE with methylene blue or indigo carmine vs. VCE (i-SCAN 2–3)	The diagnostic yield for neoplastic lesions (polypoid, non-polypoid, and CRC) was similar in the three groups (WLE 18.9%, DCE 17.8%, VCE 11.1%; *p* = 0.91).
Yang et al. [49]	Multicenter prospective randomized controlled trial	210 UC. Randomization 1:1 to: HD-DCE with targeted biopsies (*n* = 108) HD-WLE with targeted plus random biopsies (*n* = 102)	WLE vs. DCE with methylene blue or indigo carmine	HD-WLE and DCE achieved similar colitis-associated dysplasia detection rate (5.6% vs. 3.9% respectively, *p* = 0.749).

Abbreviations: CRC, colorectal cancer; HD-WLE, high-definition white light endoscopy; HD-DCE, high-definition dye chromoendoscopy; HD-VCE, high-definition virtual chromoendoscopy; IBD, inflammatory bowel disease; SD-WLE, standard-definition white light endoscopy; UC, ulcerative colitis.

**Table 2:** Studies comparing surveillance colonoscopy using virtual chromoendoscopy versus white light or dye chromoendoscopy.

Authors	Study Design	Methods	Colonoscope Technique	Results
Leifeld et al. [50]	Multi-center prospective randomized study	159 UC. Subjects underwent two colonoscopies (HD-WLE and HD-VCE) in a randomized sequence.	WLE vs. VCE (NBI)	NBI detected a comparable number of intraepithelial neoplasia to HD-WLE (31 vs. 26, *p* = 0.888). Considering only targeted biopsies in both groups, NBI showed a trend of more detection of dysplasia (1.6 times) than HD-WLE (24 vs. 15, *p* = 0.175).
Kandiah et al. [51]	Multi-center randomized controlled trial	188 IBD. Randomization 1:1 to HD-VCE (*n* = 94) or HD-WLE (*n* = 94) with targeted plus random biopsies in both arms	WLE vs. VCE (i-SCAN OE mode 2)	No difference was observed in the neoplasia detection between the two techniques (VCE 14.9% vs. WLE 24.2%; *p* = 0.14).
Bisschops et al. [52]	Multi-center randomized controlled trial	131 UC. Randomization 1:1 to HD-VCE (*n* = 65) or HD-DCE (*n* = 66) with targeted biopsies in both arms	VCE (NBI) vs. DCE with methylene blue	No difference was found in the detection of colitis-associated neoplasia between DCE and NBI [21.2% vs. 21.5%; OR 1.02 (95% CI 0.44–2.35, *p* = 0.964)].
Gulati et al. [53]	Single-center randomized crossover trial	48 IBD. Subjects underwent two colonoscopies (HD-DCE and HD-VCE) in a randomized sequence (1:1).	VCE (FICE) vs. DCE with indigo carmine	The diagnostic accuracy for the diagnosis of dysplasia applying DCE or VCE was respectively 76.9% vs. 93.7%; DCE missed 2 dysplastic lesions (18.2%) while VCE 1 dysplastic lesion (9.1%) [OR 2.0 (95% CI 0.10 to 118.0)].
González-Bernardo et al. [54]	Single-center prospective randomized study	129 IBD. Randomization 1:1 to HD-VCE (*n* = 62) or HD-DCE (*n* = 67) with targeted biopsies in both arms.	VCE (i-SCAN 1) vs. DCE with indigo carmine	No difference in the rate of detection of neoplastic lesions was observed between the two techniques (DCE 17.9% vs. VCE 11.3%; *p* = 0.2).
López-Serrano et al. [55]	Single-center retrospective case-control study	191 IBD. Comparison of HD-DCE (*n* = 98) vs. HD-VCE (*n* = 93) with targeted biopsies in both groups.	VCE (i-SCAN twin-mode 1–3) vs. DCE with indigo carmine	No significant difference in dysplasia detection was observed in the per lesion (DCE 14.6% vs. VCE 15.6%, *p* = 0.526) and per patient analysis (DCE 12.2% vs. VCE 9.7%, *p* = 0.647).

Abbreviations: FICE, flexible spectral imaging color enhancement; HD-WLE, high-definition white light endoscopy; HD-DCE, high-definition dye chromoendoscopy; HD-VCE, high-definition virtual chromoendoscopy; NBI, narrow band imaging; IBD, inflammatory bowel disease; UC, ulcerative colitis.

**Table 3:** Studies evaluating the role of random biopsies in IBD surveillance colonoscopy using HD systems.

Authors	Study Design	Methods	Colonoscope Technique	Results
Bopanna et al. [56]	Single-center prospective randomized study	28 UC. HD-WLE with 4 quadrantic random biopsies every 10 cm.	HD-WLE	No dysplasia was found in the 924 biopsy samples. Indefinite for dysplasia was observed in only seven biopsies (0.7%).
Gasia et al. [57]	Single-center retrospective audit	454 IBD. Assessing the role of surveillance strategies: SD-WLE, HD-WLE, VCE, DCE, random plus targeted biopsies, targeted biopsies only.	SD-WLE, HD-WLE, VCE (iSCAN) DCE with methylene blue or indigo carmine	Targeted biopsies with HD systems achieved a higher neoplasia diagnostic yield than random plus targeted biopsies with HD systems (respectively 19.1% vs. 10.4%, *p* = 0.02).
Watanabe et al. [58]	Multi-center randomized controlled trial	221 UC. Randomization 1:1 to HD-WLE with targeted plus random biopsies (*n* = 107) and HD-WLE with only targeted biopsies (*n* = 114).	HD-WLE in the majority of cases	Targeted biopsies group detected a similar amount of neoplasia compared to random plus targeted biopsies group (respectively 11.4% vs. 9.3%; *p* = 0.617).
Hata et al. [59]	Multi-center retrospective cohort study	195 UC. Comparing long-term efficacy of targeted vs. targeted plus random biopsies using follow-up data of the Watanabe et al. trial.	HD-WLE in the majority of cases	The likelihood to develop CRC in subjects with a negative examination was low (Invasive CRC: 0.77 per 1000 patient-years. Advanced neoplasia HGD/CRC-TIS: 2.3 per 1000 patient-years).
Moussata et al. [60]	Multi-center prospective cohort study	1000 IBD. Evaluation of the role of additional random biopsies in HD-DCE.	HD-DCE with indigo carmine	Random biopsies exhibited a low dysplasia diagnostic yield (0.2% per biopsy, 68/31865). Personal history of neoplasia, tubular colon, and PSC were independently associated with the detection of dysplasia by random biopsies.
Hu et al. [61]	Multi-center retrospective study	300 IBD contributing 442 colonoscopes with detection of dysplasia. Determination of the additional dysplasia diagnostic yield by random biopsies in HD-WLE and HD-DC.	HD-WLE and HD-DCE	Dysplasia discovered by random biopsies was linked to longer disease duration (OR 1.04, 95% CI 1.01–1.07), active inflammation (OR 2.89, 95% CI 1.26–6.67), PSC (OR 3.66, 95% CI 1.21–11.08).

Abbreviations: CRC, colorectal cancer; HD-WLE, high-definition white light endoscopy; HD-DCE, high-definition dye chromoendoscopy; HD-VCE, high-definition virtual chromoendoscopy; IBD, inflammatory bowel disease; SD-WLE, standard-definition white light endoscopy; UC, ulcerative colitis.

## Data Availability

No new data were generated or analyzed in support of this research.
